# Circulating Bacterial DNA as a Novel Blood-Based Biomarker in Type 2 Diabetes Mellitus (DM2): Results from the PROMOTERA Study

**DOI:** 10.3390/ijms26146564

**Published:** 2025-07-08

**Authors:** Robertina Giacconi, Patrizia D’Aquila, Fabiola Olivieri, Davide Gentilini, Luciano Calzari, Carlo Fortunato, Gretta Veronica Badillo Pazmay, Mirko Di Rosa, Giada Sena, Elisabetta De Rose, Antonio Cherubini, Riccardo Sarzani, Roberto Antonicelli, Giuseppe Pelliccioni, Anna Rita Bonfigli, Roberta Galeazzi, Fabrizia Lattanzio, Giuseppe Passarino, Dina Bellizzi, Francesco Piacenza

**Affiliations:** 1 Advanced Technology Center for Aging Research, Istituto di Ricovero e Cura a Carattere Scientifico (IRCCS) and Istituto Nazionale di Ricerca e Cura Anziani (INRCA), 60127 Ancona, Italy; r.giacconi@inrca.it (R.G.); f.olivieri@staff.univpm.it (F.O.); c.fortunato2@inrca.it (C.F.); g.badillo@inrca.it (G.V.B.P.); f.piacenza@inrca.it (F.P.); 2 Department of Biology, Ecology and Earth Sciences, University of Calabria, 87036 Rende, Italy; patrizia.daquila@unical.it (P.D.); giada.sena@unical.it (G.S.); elisabetta.derose@unical.it (E.D.R.); giuseppe.passarino@unical.it (G.P.); 3 Department of Clinical and Molecular Sciences, Disclimo, Università Politecnica delle Marche, 60127 Ancona, Italy; a.cherubini@inrca.it (A.C.); riccardo.sarzani@gmail.com (R.S.); 4 Department of Brain and Behavioral Sciences, University of Pavia, 27100 Pavia, Italy; 5 Bioinformatics and Statistical Genomics Unit, Istituto Auxologico Italiano IRCCS, 20095 Cusano Milanino, Italy; luciano.calza@gmail.com; 6 Unit of Geriatric Pharmacoepidemiology and Biostatistics, Istituto di Ricovero e Cura a Carattere Scientifico (IRCCS) and Istituto Nazionale di Ricerca e Cura Anziani (INRCA), 60124 Ancona, Italy; m.dirosa@inrca.it; 7 Geriatria, Accettazione Geriatrica e Centro di Ricerca per L’invecchiamento, Istituto di Ricovero e Cura a Carattere Scientifico (IRCCS) and Istituto Nazionale di Ricerca e Cura Anziani (INRCA), 60127 Ancona, Italy; 8 Internal Medicine and Geriatrics, Istituto di Ricovero e Cura a Carattere Scientifico (IRCCS) and Istituto Nazionale di Ricerca e Cura Anziani (INRCA), 60124 Ancona, Italy; 9 Cardiology Unit, Istituto di Ricovero e Cura a Carattere Scientifico (IRCCS) and Istituto Nazionale di Ricerca e Cura Anziani (INRCA), 60124 Ancona, Italy; r.antonicelli@inrca.it; 10 Neurology Unit, Istituto di Ricovero e Cura a Carattere Scientifico (IRCCS) and Istituto Nazionale di Ricerca e Cura Anziani (INRCA), 60124 Ancona, Italy; g.pelliccioni@inrca.it; 11 Scientific Direction, Istituto di Ricovero e Cura a Carattere Scientifico (IRCCS) and Istituto Nazionale di Ricerca e Cura Anziani (INRCA), 60124 Ancona, Italy; a.bonfigli@inrca.it (A.R.B.); f.lattanzio@inrca.it (F.L.); 12 Clinic of Laboratory and Precision Medicine, Istituto di Ricovero e Cura a Carattere Scientifico (IRCCS) and Istituto Nazionale di Ricerca e Cura Anziani (INRCA), 60124 Ancona, Italy; r.galeazzi@inrca.it

**Keywords:** bacterial DNA, epigenetic markers, diabetes, inflammation, diabetic nephropathy

## Abstract

Blood bacterial DNA (BB-DNA) has been identified as a novel biomarker for metabolic dysfunction, yet its relationship with epigenetic features in type 2 diabetes mellitus (DM2) patients remains largely unexplored. This study investigated the relationship between BB-DNA and epigenetic, inflammatory, and aging-related markers in 285 elderly both with and without DM2. BB-DNA levels were higher in DM2 patients than in non-diabetic subjects, with the highest levels in those with severe renal impairment. BB-DNA showed a positive association with plasma IL-1β, linking bacterial DNA to systemic inflammation. Epigenetic analysis revealed a negative correlation between BB-DNA and DNA methylation-based leukocyte telomere length, suggesting accelerated aging in DM2. Additionally, BB-DNA was positively associated with DNAm-based biological age estimators, particularly DNAmPhenoAge and DNAmAge Skin Blood Clock. BB-DNA also correlated with DNAmVEGFA and DNAmCystatin C, key markers of diabetic nephropathy and vascular dysfunction. Furthermore, BB-DNA levels were associated with hypomethylation of genes involved in inflammation (e.g., *IL1β*, *TNFα*, *IFNγ*), cellular senescence (*p16*, *p21*, *TP53*), and metabolic regulation (e.g., *IGF1*, *SREBF1*, *ABCG1*, *PDK4*). These associations suggest that increased BB-DNA may reflect and potentially promote a pro-inflammatory and pro-senescent epigenetic profile in DM2. Importantly, many of these associations remained significant after adjusting for diabetes status, supporting BB-DNA as a robust biomarker across clinical subgroups. These findings provide new insights into the relationship between BB-DNA, inflammation, and epigenetic aging in DM2, highlighting BB-DNA as a potential biomarker for disease progression and complications, particularly in relation to renal dysfunction and systemic inflammation.

## 1. Introduction

Type 2 diabetes mellitus (DM2) represents a major public health challenge, particularly in developed countries, where older adults constitute the most number of affected patients [1,2]. Aging impairs insulin secretion from β cells, reduces insulin sensitivity, and promotes β-cell death by inducing mitochondrial dysfunction. In older subjects, abnormalities in both insulin sensitivity and insulin secretion gradually lead to impaired glucose tolerance and consequently to clinically manifest diabetes. Postprandial hyperglycemia is a hallmark of DM2 in older patients [3]. Moreover, DM2 in old age is associated with usual complications including micro and macro vascular disease and it is related to various other comorbidities and geriatric conditions, including cognitive impairment, urinary incontinence, sarcopenia, and increased risk of falls [4,5]. An overall state of chronic inflammation and dysregulated immune system may underlie these increased risks [6]. Among the key mechanisms found to be altered in DM2 patients, epigenetic modifications, especially DNA methylation, where methyl groups are added to DNA molecules affecting gene expression without altering the DNA sequence, are emerging as crucial links between genetic, environmental, and lifestyle factors in DM2 development and progression.

Epigenetic alterations are acquired during aging through both intrinsic processes, driven by stochastic and genetic influences, and extrinsic factors, such as environmental exposures and exogenous stressors. A dysregulation of epigenetic modifications may contribute to the development of various age-related diseases [7]. Age-related changes in DNA methylation levels have been well documented, with evidence indicating a strong correlation between chronological age and methylation alterations at approximately one-third of methylation sites in peripheral blood samples [8]. Based on this observation, the measurement of epigenetic modifications has been increasingly integrated into powerful analytical approaches known as epigenetic clocks. These clocks are attracting significant attention for their ability to bridge the gap between biological and chronological age and to assess the efficacy of aging interventions in a personalized and disease-specific manner [9,10,11]. Epigenetic clocks are mathematically derived estimators that utilize methylation values at specific CpG sites across the genome. They are widely applied to determine the biological age of tissues and cells, offering valuable insights into aging processes and age-related diseases. Of the first-reported clocks, the well-known Horvath clock was constructed by analyzing multiple tissues and including the blood data from Hannum et al. [12,13]. Among the numerous epigenetic clocks, GrimAge and PhenoAge were developed using physiological variables to capture age-related physiological dysregulation and have been linked to various age-associated diseases [14]. Several studies have reported associations between epigenetic age acceleration metrics, such as GrimAA and PhenoAA, and DM2, as well as glycemic traits [15,16]. Beyond epigenetic clocks, epimutations have been proposed as adaptive responses to environmental stimuli. Notably, microbial interactions can influence a wide range of epigenetic mechanisms, including DNA methylation, histone modifications, chromatin-associated complexes, and non-coding RNAs, ultimately altering chromatin structure and gene expression [17]. There is substantial evidence that epigenetic mechanisms play a critical role in regulating host–pathogen interactions, with bacterial infections inducing epigenetic modifications that can alter the host phenotype.

Recent studies have explored the presence of bacterial DNA in the bloodstream and its potential contribution to disease pathogenesis [18]. In fact, bacterial DNA and culturable bacteria have been found in the blood of healthy subjects and patients with non-infectious diseases, such as diabetes, cardiovascular disease, Alzheimer’s disease, and colorectal cancer [19,20,21,22,23]. Several studies have highlighted its potential as a non-invasive biomarker for enhancing diagnosis and treatment effectiveness. The function and origin of blood bacterial DNA (BB-DNA) are still debated, but a series of studies have identified members of various microbiome niches, including the gut, oral cavity, airways, and skin, although more evidence is required to confirm that healthy human blood contains living and active microbiota. BB-DNA can enter the bloodstream due to increased intestinal permeability (“leaky gut”), triggering systemic inflammation and metabolic dysfunction [22,23]. Despite growing evidence of BB-DNA’s role in inflammation and metabolic dysfunction, its association with epigenetic features in older adults with DM2 remains unexplored.

In this study, we aim to bridge this knowledge gap by quantifying BB-DNA in the bloodstream of 285 older adult patients, both with and without DM2, who were hospitalized at the Istituto di Ricovero e Cura a Carattere Scientifico (IRCCS) and Istituto Nazionale di Ricerca e Cura Anziani (INRCA). Our aim was to evaluate its association with various epigenetic DNA features, including DNA methylation-based estimators of age, telomere length, and angiogenic and pro-inflammatory factors.

## 2. Results

### 2.1. Clinical Characteristics of the PROMOTERA Cohort

Table 1 provides an overview of the main characteristics of the studied cohort.

Patients (ND) with other diseases and no evidence of type 2 diabetes mellitus (DM2) had a similar age and sex distribution to those with DM2. However, DM2 patients exhibited a higher prevalence of atrial fibrillation (AF), ischemic heart disease (IHD), chronic heart failure (CHF), chronic kidney disease (CKD), and stroke. In addition, DM2 patients showed elevated neutrophil counts, fasting glucose levels, and decreased estimated glomerular filtration rates, as well as an increasing trend in inflammatory markers, such as the neutrophil-to-lymphocyte ratio (NLR) and the systemic inflammatory response index (SIRI). We observed that BB-DNA levels were more elevated in DM2 patients with respect to ND subjects (Table 1). The increase is especially evident in those patients with severe renal impairment compared to DM2 patients with mild or moderate CKD, as well as ND patients, regardless of the CKD stage (Figure S1). Moreover, BB-DNA levels were analyzed in relation to other DM2 complications, including ischemic heart disease (IHD), cerebrovascular disease (CeVD), peripheral neuropathy (PN), and peripheral artery disease (PAD). Notably, DM2 patients exhibited significantly higher BB-DNA levels in the presence of IHD, PN, and PAD compared to those without these conditions, while no significant difference was observed for CeVD after multiple adjustments (Figure S2, Supplementary Material).

### 2.2. Association of BB-DNA with Plasma IL-1β Levels

**Figure 1:**
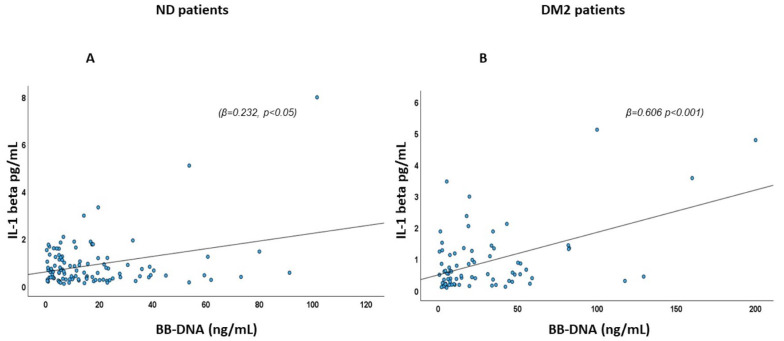
Scatter plots and linear regression between BB-DNA and IL1-β. A positive correlation between BB-DNA and plasma IL-1β levels was observed in (**A**) ND (β = 0.232, *p* < 0.05) and (**B**) DM2 patients (β = 0.606 *p* < 0.001) and after correction with age, sex, atrial fibrillation (AF), ischemic heart disease (IHD), chronic heart failure (CHF), chronic kidney disease (CKD), stroke, neutrophil count, and estimated glomerular filtration rate (eGFR).

The relationship between BB-DNA and plasma IL-1β levels was evaluated using linear regression, with age and sex, AF, IHD, CHF, CKD, stroke, neutrophil count, and eGFR included as covariates. A significant positive association was observed between BB-DNA and the pro-inflammatory cytokine in both DM2 patients (β = 0.606, *p* < 0.001) and ND patients (β = 0.232, *p* < 0.05) (Figure 1). A sensitivity analysis using the SIRI index instead of neutrophil count confirmed the association between IL-1β and BB-DNA in both ND patients (β = 0.362, *p* < 0.01) and DM2 patients (β = 0.567, *p* < 0.001).

### 2.3. Association of BB-DNA and DNA Methylation-Based Estimator of Leukocyte Telomere Length (DNAm-LTL)

**Figure 2:**
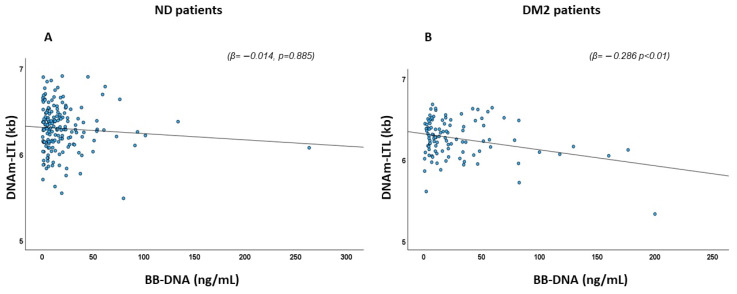
Scatter plots and linear regression between BB-DNA and DNAm-LTL in (**A**) ND and (**B**) DM2 patients. A negative association between BB-DNA and DNAm-LTL was observed in DM2 patients (β = −0.286 *p* < 0.01), but not in ND patients (β = −0.014, *p* = 0.885) after correction with age, sex, atrial fibrillation (AF), ischemic heart disease (IHD), chronic heart failure (CHF), chronic kidney disease (CKD), stroke, neutrophil count, and estimated glomerular filtration rate (eGFR).

The association between BB-DNA and the DNA methylation-based estimator of leukocyte telomere length (DNAm-LTL) [24] was assessed using linear regression with age, sex, AF, IHD, CHF, CKD, stroke, neutrophil count, and estimated glomerular filtration rate (eGFR) included as covariates. A significant negative association between DNAm-LTL and BB-DNA was observed in DM2 patients (β = −0.286, *p* < 0.01), whereas no such association was found in ND patients (β = −0.014, *p* = 0.885) (Figure 2).

### 2.4. Analysis of the Association of BB-DNA and DNAm Biological Age Estimators

To investigate whether BB-DNA and DNAm-based estimates of biological age are associated in our cohort, we applied several models (Table 2).

Three epigenetic biomarkers exhibited a significant positive association with BB-DNA in DM2 patients, but not in ND patients. Specifically, DNAm PhenoAge, an epigenetic clock that integrates chronological age with DNAm-based estimates of biochemical and hematological variables, along with EpigeneticAge by Zhang, DNAmAge Skin Blood Clock, showed positive associations. DNAmAge Hannum displayed a trend toward association, while the classical DNAmAge and algorithms linked to lifespan, such as DNAm GrimAge and DNAm GrimAge2, did not show any significant association with BB-DNA. Regarding the difference between an individual’s epigenetic age and their chronological age, as measured by age acceleration, a positive association was observed between Age Acceleration PhenoAge and BB-DNA, with a trend toward association for Age Acceleration Residual Hannum (Table 2). However, no association with BB-DNA was found for Age Acceleration Grimage, IEAA (Intrinsic Epigenetic Age Acceleration) or IEAA Hannum, which is derived specifically from the blood-specific Hannum epigenetic clock.

### 2.5. Association Between BB-DNA and DNAm-Based Estimators of Angiogenic, Pro-Inflammatory Factors, and DNAm-Based Estimators of Blood Cell Types

**Figure 3:**
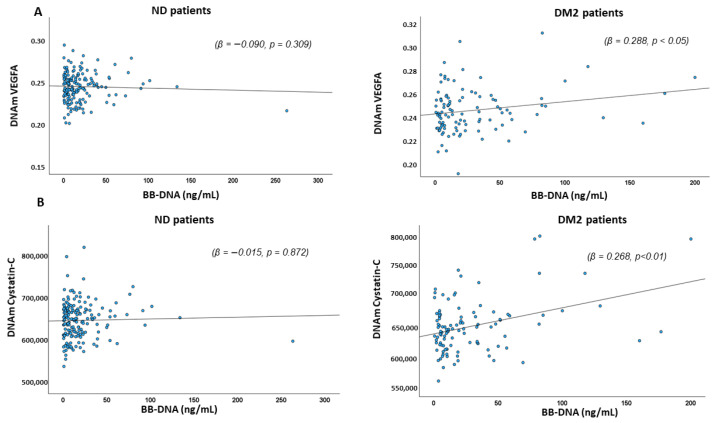
Scatter plots and linear regression between BB-DNA and DNAmVEGFA (**A**) and DNAm cystatin C (**B**). A significant positive association between BB-DNA and plasma DNAmVEGFA (β = 0.288, *p* < 0.05) and DNAmCystatin C (β = 0.268, *p* < 0.01) was observed in DM2 patients, whereas no association was found in ND patients (β = −0.090, *p* = 0.309 and β = −0.015, *p* = 0.872, respectively) after adjusting for age, sex, atrial fibrillation (AF), ischemic heart disease (IHD), chronic heart failure (CHF), chronic kidney disease (CKD), stroke, neutrophil count, and estimated glomerular filtration rate (eGFR).

**Figure 4:**
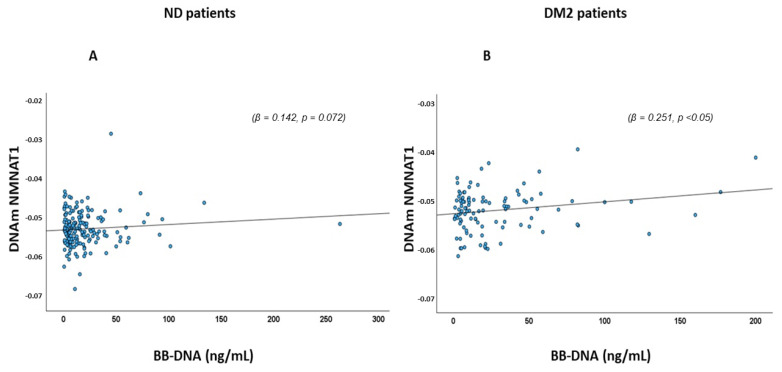
Scatter plots and linear regression between BB-DNA and DNAmNMNAT1 in (**A**) ND and (**B**) DM2 patients. A significant positive correlation was observed between BB-DNA and plasma DNAmNMNAT1 in DM2 patients (β = 0.251, *p* < 0.05), whereas no association was found in ND patients (β = 0.142, *p* = 0.072) after adjusting for age, sex, atrial fibrillation (AF), ischemic heart disease (IHD), chronic heart failure (CHF), chronic kidney disease (CKD), stroke, neutrophil count, and estimated glomerular filtration rate (eGFR).

The relationship between BB-DNA and various DNAm markers, including DNAmVEGFA, DNAmCystatin C, DNAmNMNAT1, and other pro-inflammatory factors such as DNAmCXCL9, DNAmCXCL10, DNAmCXCL11, DNAmCCL11, DNAmBeta-2-Microglobulin, DNAmIL-18R1, DNAmIL-6, DNAmOncostatin M, DNAmTNF-β, DNAmGDF-15, DNAmPAI-1, DNAmTIMP1, and DNAmLeptin was assessed. In DM2, a significant positive association of DNAmVEGFA and DNAmCystatin C (β = 0.288, *p* < 0.05; β = 0.268, *p* < 0.01, respectively) emerged, while no such association was found in ND patients (β = −0.090, *p* = 0.309; β = −0.015, *p* = 0.872, respectively) (Figure 3A,B). Similarly, DNAmNMNAT1 showed a positive correlation with BB-DNA in DM2 patients (β = 0.251, *p* < 0.05), with a slight association in ND patients (β = 0.142, *p* = 0.072) (Figure 4).

No significant associations were identified for the remaining inflammatory markers in either DM2 or ND patients (Table S1). No difference in the DNAm-based estimators of blood cell types was observed in the PROMOTERA cohort (Table S2) except for CD8 naive cells, which showed a negative association with BB-DNA (β = −0.225, *p* < 0.05). Stepwise linear regression analysis was also performed to evaluate the association of BB-DNA with the beta values of the main inflammatory genes in DM2 patients (*IFNγ*, *TNFα*, *IL1β*, *IL6*, *IL10*, *CRP*, and *NFKB1*) (Table 3). Most CpG sites showed a negative association with BB-DNA, suggesting that elevated BB-DNA levels are linked to hypomethylation of these genes.

Table 3 also displays the key CpG sites of senescent marker genes (*CDKN1A/p21*, *CDKN2A/p16* and *TP53*) associated with BB-DNA. Most of these sites exhibited a negative association after stepwise linear regression, indicating that elevated BB-DNA levels are correlated to hypomethylation, which may lead to an increase in the expression of these senescence markers in blood cells.

### 2.6. Association Between BB-DNA and Epigenetic Signatures of Type 2 Diabetes Mellitus

Major differentially methylated genes in DM2 were identified [30,31] and the association between their beta values and BB-DNA was evaluated using stepwise linear regression, adjusted for sex and age, AF, IHD, CHF, CKD, stroke, neutrophil count, and eGFR (Table 4).

The methylation of most CpG units shows a negative association with BB-DNA, indicating that an increase in bacterial DNA is related to the hypomethylation of these genes, suggesting their enhanced expression. Stepwise linear regression in ND patients revealed no association between BB-DNA and CpG sites in the *PDK4*, *POP7*, *DECR2*, *TCEB2*, or *PRDX5* genes. However, regarding other genes, a correlation was identified with distinct CpG sites compared to those observed in DM2 patients (Table S3), except for ARRDC4_cg09442792, SREBF1_cg27407935, BSN_cg13444307, and BSN_cg19602139, which are associated with BB-DNA in both patient groups.

### 2.7. Associations Between BB-DNA and Epigenetic Markers in the Overall Cohort

To assess whether the associations observed in DM2 and ND groups were consistent in the combined population, additional analyses were performed including diabetes status as a covariate in the regression models. In the entire PROMOTERA cohort, several associations remained statistically significant. Specifically, BB-DNA was positively associated with IL-1β (β = 0.252 *p* < 0.01, Figure S3, Supplementary Material), negatively associated with DNAm-LTL (β = –0.143, *p* = 0.013; Figure S4, Supplementary Material), and positively associated with DNAm-NMNAT1 (β = 0.195, *p* < 0.01; Figure S5 Supplementary Material). A trend toward positive association was also observed for DNAm-VEGFA (β = 0.085, *p* = 0.121) and DNAm-cystatin C (β = 0.173, *p* = 0.072) (Figure S5, Supplementary Material). DNAmAge Skin Blood Clock remained significantly associated with BB-DNA in the combined cohort (Table S4, Supplementary Material). Moreover, stepwise linear regression analyses performed in the overall population confirmed several significant CpG sites for inflammatory mediators and senescence-associated genes, including CDKN1A/p21, CDKN2A/p16, and TP53 (Table S5, Supplementary Material). In addition, in the full cohort, multiple CpG sites within genes previously implicated in the epigenetic signature of DM2 showed significant associations with BB-DNA levels (Table S6, Supplementary Material). Finally, the associations between BB-DNA and both DNAm-based estimators of blood cell composition and additional pro-inflammatory factors in the overall PROMOTERA cohort were consistent with the results obtained in the separate DM2 and ND groups (Tables S7 and S8, Supplementary Material).

## 3. Discussion

Growing evidence is demonstrating that the levels of blood bacterial DNA (BB-DNA) represent a biomarker for human health. High BB-DNA levels have been linked to geographic origin, behavior habits, and the onset of various pathological conditions, including diabetes, has been proven [19,21,22,32,33,34]. Moreover, DNA methylation signatures have been extensively studied, leading to the development of several models for various tissues that identify DNAm-based biomarkers associated with aging and age-related diseases [35].

Having found high levels of BB-DNA in older DM2 subjects in this study, as well as their positive association with IL-1β, a pro-inflammatory cytokine that modulates insulin secretion and β-cell apoptosis, we explored the potential relationship between BB-DNA and epigenetic biomarkers characteristic of DM2.

This study is the first to report the association between BB-DNA with DNAm-LTL [24] and accelerated epigenetic aging in DM2 patients, suggesting a heightened risk of developing DM2-related complications and increased mortality over time [36,37]. Notably, both telomere length (TL) and DNAm-LTL, particularly the latter, even more than directly measured TL, are widely recognized as inversely associated with aging and the incidence, progression, and mortality of several age-related diseases, including DM2 [24,37,38]. The negative association we observed between BB-DNA and DNAm-LTL, an epigenetic biomarker that reflects not only the replicative history of cells but also serves as a valuable marker of age-related pathologies, aligns with previous evidence suggesting accelerated biological aging during infections [39].

Notably, only DNAm-based estimators of biological age showed a positive association with BB-DNA levels, indicating that BB-DNA is not correlated with chronological age but rather with an individual’s biological age. This finding aligns with existing evidence, reinforcing the idea that high BB-DNA levels are not merely a consequence of aging but are instead linked to overall health status. Indeed, elevated BB-DNA levels have been observed in diabetic patients with severe renal dysfunction, further supporting its potential role as a marker of disease severity and characterized by an elevated inflammatory state. More specifically, high BB-DNA levels could be considered a biomarker of diabetic nephropathy, as they have been associated with increased methylation levels of VEGFA and NMNAT1, key contributors to the progression of diabetic kidney disease. Evidence has demonstrated that in diabetes, VEGFA is initially upregulated in both the glomeruli and the tubular compartment, in which it induces vascular remodeling, inflammatory processes, glomerulosclerosis, and tubulointerstitial fibrosis; its expression then dramatically drops as the disease progresses, leading to endothelial apoptosis and proteinuria [40]. VEGFA also plays an essential role in diabetic nephropathy. Similarly, the reduction in NMNAT1 expression, by unbalancing NAD+ homeostasis, causes mitochondrial dysfunction, is associated with insulin resistance, and impairs hepatic insulin signaling and hepatokine expression [41]. The increase in BB-DNA has been associated with inflammation and tissue damage, particularly in the presence of CKD [42]. In this context, the positive correlation between BB-DNA and DNAmNMNAT1 could be associated with mitochondrial damage. The hypothesis suggests that inflammation or damage induced by BB-DNA may contribute to mitochondrial dysfunction and insulin resistance through the down-expression of NMNAT1 driven by its gene hypermethylation [43]. Additionally, high levels of BB-DNA correlate with DNAm cystatin C, a well-established proxy for serum cystatin C levels and, thus, a biomarker of the glomerular filtration rate and all-cause mortality risk [44].

Therefore, BB-DNA being positively correlated with VEGFA and cystatin C could promote inflammation, vascular damage, and renal complications. Notably, our analysis revealed that the increase in BB-DNA in relation to CKD severity was observed exclusively in patients with DM2, while in non-diabetic individuals BB-DNA levels remained comparable across CKD stages. This pattern suggests a synergistic interaction between diabetes and renal dysfunction that may amplify gut permeability [45], systemic inflammation, and the accumulation of circulating bacterial products. In DM2 patients, these processes are likely exacerbated by chronic hyperglycemia, immune dysregulation, and gut microbiota alterations, resulting in higher BB-DNA levels in advanced CKD. Conversely, in non-diabetic individuals, renal impairment alone may be insufficient to elicit a comparable increase in BB-DNA levels, possibly due to preserved gut barrier function and lower baseline inflammation. These findings support the view that BB-DNA may not only serve as a biomarker of diabetic nephropathy, but also reflect the broader pathophysiological interplay between metabolic dysregulation and renal decline in older adults with diabetes. Furthermore, the observed association between elevated BB-DNA levels and specific diabetic complications, such as IHD, PN, and PAD, but not CeVD, strengthens the hypothesis that BB-DNA may serve not only as a marker of cardiovascular and peripheral vascular involvement, but also as an indicator of disease severity in DM2.

Consistent with the existing literature, increased BB-DNA levels were associated with inflammation and tissue damage, as indicated by elevated plasma IL-1β and the hypomethylation of key inflammatory genes, including *INFγ*, *TNFα*, *NFKB*, and *CRP*. The consequent overexpression of these genes may contribute to the establishment of the chronic inflammatory state that characterizes diabetes [29,30]. This finding is further supported by the observed hypomethylation of key senescence markers p21, p16, and p53, which are known to drive inflammation and cellular senescence by suppressing *DNMT1* expression in the tubular compartment, a process mediated by persistent hyperglycemia [46]. In this context, neutrophils may play a pivotal role in sustaining chronic inflammation in DM2, as key effectors of the innate immune barrier in the intestinal mucosa. They are the first leukocytes to be mobilized and recruited to sites of inflammation [47]. Alterations in gut microbiota composition can lead to increased neutrophil recruitment and activation, leading to enhanced production of neutrophil extracellular traps (NETs) through phagocytosis and degranulation, ultimately contributing to disease progression and systemic inflammation [48,49]. These mechanisms may contribute to the neutrophil-driven inflammatory profile observed in DM2 patients and support the hypothesis of a gut-mediated innate immune activation contributing to elevated BB-DNA levels and chronic inflammation. However, whether this chronic inflammatory state is primarily driven and sustained by elevated BB-DNA levels or if BB-DNA accumulation is a consequence of pre-existing inflammation, remains an open question that warrants further investigation.

Finally, the association between BB-DNA and the methylation of CpG sites in key diabetes-related genes suggests that BB-DNA might contribute to a widespread remodeling of methylation profiles in genes strictly involved in the pathology. With a few exceptions, a general trend of hypomethylation was observed in genes involved in glucose metabolism, insulin resistance (*IGF1*, *FAM3C*, *OAZ2*, *PLAGL1*, *SREBF1*, *ARRDC4*, *PFKFB2*, *PDK4*), and lipid metabolism (*DECR2*, *ABCG1*, *PDK4*), as well as in the processes of transcription and cellular senescence (*UFM1*, *TCEB2*). While a causal role of BB-DNA in the onset or progression of diabetes and its renal complications cannot be established, targeted studies are needed to clarify these mechanisms.

Despite the relatively small sample size, this study is particularly significant as it evaluates the most relevant epigenetic clock estimates in relation to BB-DNA levels, providing insight into the potential of this biomarker for assessing biological aging and disease progression. Importantly, the consistency of several associations was confirmed in the overall PROMOTERA cohort when diabetes status was included as a covariate, further supporting the robustness and generalizability of our findings across clinical subgroups. However, several limitations should be considered. First, its cross-sectional design prevents establishing a causal relationship between BB-DNA and epigenetic, inflammatory, and metabolic markers. Second, the relatively small sample size may limit the generalizability of the findings. Third, although adjustments were made for major comorbidities, other potential confounders such as medication use, gut microbiota composition, diet, and lifestyle factors were not accounted for. Fourth, BB-DNA quantification was performed using PCR, which does not distinguish between bacterial fragments and viable bacteria. Finally, the significant difference in atrial fibrillation prevalence (51.4% in DM2 vs. 0% in controls) could have influenced inflammatory markers and BB-DNA levels, potentially affecting the interpretation of the results.

This study highlights the potential of BB-DNA presence in the blood as an inexpensive, non-invasive, rapid, and easily accessible tool for estimating its role as a biomarker in DM2 progression in older individuals.

## 4. Materials and Methods

### 4.1. Study Population, Recruitment, Data, and Blood Collection

Two hundred and eighty five patients were identified from the PROMOTERA study (CE INRCA 20031, 4 February 2021) within the Report-AGE project based on the availability of blood samples and complete clinical information. The patients were categorized into two groups: 107 individuals with DM2 and 178 with other diseases and no evidence of DM2 (ND) and matched for age and sex. The Report-AGE project, a large-scale observational study of older patients (age >65 years) hospitalized at IRCCS INRCA between 16 June 2012, and 3 November 2017, is registered under Trial Registration no. NCT01397682 [50]. Blood samples, collected in EDTA tubes (Becton, Dickinson and Company, Franklin Lakes NJ, USA) within the initial 24 h following hospital admission, were stored in the INRCA BioGer Biobank. Comprehensive data on the medical history of all participants in this study were retrieved from medical records. Diagnoses were coded in accordance with the International Classification of Diseases, 9th revision (http://www.icd9data.com/, accessed in 1 June 2025). Hypertension was defined as systolic blood pressure ≥140 mmHg and/or diastolic blood pressure ≥ 90 mmHg, in accordance with the current guidelines for the diagnosis and management of high blood pressure. Blood admission, laboratory parameters such as blood cell counts, creatinine, albumin, hemoglobin, and aspartate aminotransferase/alanine aminotransferase (AST/ALT) were measured using standardized procedures. The SIRI index was calculated as the formula of neutrophil count × monocyte count/lymphocyte count. GFR was estimated according to the Chronic Kidney Disease Epidemiology Collaboration (CKD-EPI) equation [51].

### 4.2. DNA Extraction and Methylation Assay

Genomic DNA was extracted from whole blood using the QIAamp DNA Blood Kit (Qiagen GmbH, Hilden, Germany) and bisulfite-converted using the EZ DNA Methylation Kit (Zymo Research, Freiburg, Germany). Genome-wide DNA methylation was assessed with the Infinium Human Methylation EPIC BeadChip (Illumina, Berlin Germany), following the manufacturer’s instructions [52]. RStudio software (Version 2023.12.0.369 PBC, Boston, MA, USA) was used for processing and normalization of raw intensities (*. idat) using the R package “minfi” v. 1.48.0. The methylation levels obtained were stored as Beta values.

### 4.3. Blood Bacterial DNA (BB-DNA) Quantification

DNA was extracted from 300 µL of whole blood samples using the QIAamp DNA Blood mini kit (Qiagen GmbH, Hilden, Germany) according to the manufacturer’s instructions. Blood Bacterial DNA (BB-DNA) quantification was carried out by the amplification of the 16S rRNA gene as previously reported [19]. Briefly, specific universal primers targeting the V3–V4 hypervariable region of the bacterial 16S rDNA were used in real-time qPCR reactions. The PCR mixture (20 µL) consisted of 20 ng of DNA, SensiFAST SYBR Hi-ROX Mix 1× (Bioline, London, UK), and 0.4 µM of the following primers: 5′-TCCTACGGGAGGCAGCAGT-3′ and Rev 5′-GGACTACCAGGGTATCTAATCCTGTT-3′. The thermal profile used for the reaction included a heat activation of the enzyme at 95 °C for 2 min, followed by 40 cycles of denaturation at 95 °C for 15 s and annealing/extension at 60 °C for 60 s, followed by melt analysis ramping at 60–95 °C. All measurements were taken in the log phase amplification. Standard curves obtained using a 10-fold dilution series of bacterial DNA standards (Femto bacterial DNA quantification kit, Zymo Research) ranging from 0.0002 to 2 picograms were routinely run with each sample set and compared with previous standard curves to check for consistency between runs. Amplicon quality was ascertained by the melting curves. Amplifications were performed in triplicate on the QuantStudio 3 Real-Time PCR System (ThermoFisher Scientific, Segrate, Milan, Italy). BB-DNA levels were expressed as nanograms per millilitre of whole blood and were calculated by normalizing the absolute quantities of BB-DNA of each sample to their dilution factors and to the volume of starting blood used for the extraction.

### 4.4. Estimation of Surrogate Biomarkers

Whole-genome methylation data were analyzed to derive a range of DNAm-based estimates, including predictors of biological aging, biomarkers reflecting plasma protein levels, DNAm-based telomere length in leukocytes (DNAm-LTL), and inferred blood cell count estimates [24]. The surrogate biomarkers were calculated using the R package dnaMethyAge v.0.2.0, a user-friendly R package to predict epigenetic age and calculate age acceleration from DNA methylation data (https://github.com/yiluyucheng/dnaMethyAge, accessed on 1 May 2025). Beta values from CpG sites associated with *IFNγ*, *TNFα*, *IL6*, *IL1β*, *IL10*, *NFKB1*, *CRP*, *CDKN1A (p21)*, *CDKN2A (p16)*, *TP53*, *IGF1*, *BSN*, *DECR2*, *PRDX5*, *FBXO42*, *COMMD7*, *TCEB2*, *POP7*, *FAM3C*, *OAZ2*, *HEG1*, *PLAGL1*, *SREBF1*, *ARRDC4*, *PFKFB2*, *UFM1*, *ABCG1*, and *PDK4* genes were also analyzed to evaluate the relationship between their methylation levels and BB-DNA.

### 4.5. Statistical Analysis

Subject characteristics were reported as mean ± standard error of the mean (SEM) or percentages for continuous and categorical variables, respectively. For continuous variables, normal distribution was verified by the 1-sample Kolmogorov–Smirnov test. All the variables not normally distributed were log-transformed. Differences among groups were checked by One-way Analysis of Variance for continuous variables and Pearson’s χ2 test for categorical variables. The Spearman correlation coefficient was used to assess the strength and direction of the monotonic relationship between variables. A linear regression analysis using stepwise methods was performed to investigate the association between CpG methylation levels of various target genes and BB-DNA. The methylation levels of CpG sites in the following genes were analyzed: *TNFα*, *IL6*, *IL1β*, *IL10*, *NFKB1*, *CRP*, *CDKN1A (p21)*, *CDKN2A (p16)*, *TP53*, *IGF1*, *BSN*, *DECR2*, *PRDX5*, *FBXO42*, *COMMD7*, *TCEB2*, *POP7*, *FAM3C*, *OAZ2*, *HEG1*, *PLAGL1*, *SREBF1*, *ARRDC4*, *PFKFB2*, *UFM1*, *ABCG1*, and *PDK4*. Linear regression analysis using stepwise methods was also performed to assess the association between BB-DNA, DNAm-based estimators of age, telomere length, and angiogenic and pro-inflammatory factors in PROMOTERA cohort. The analysis was adjusted for the main comorbidities that differed in prevalence between the two patient groups. All analyses were performed using the SPSS/Win program (Version 29.0.1.0 (171); SPSS Inc., Chicago, IL, USA) and RStudio software (Version 2023.12.0.369 PBC, Boston, MA, USA). Two-sided *p*-values less than 0.05 were considered statistically significant, with adjustments for multiple comparisons applied as appropriate using the Bonferroni correction.

## Figures and Tables

**Table 1:** Clinical characteristics of the PROMOTERA patients.

	ND (178)	DM2 (107)	*p* Value
Age (yrs)	83.1± 0.6	83.4 ± 0.7	NS
Sex, Male *n* (%)	77 (43.3%)	53 (49.5%)	NS
Hypertension *n* (%)	137 (77.0%)	90 (84.1%)	NS
AF *n* (%)	0 (0%)	55 (51.4%)	<0.001
IHD *n* (%)	18 (10.1%)	20 (18.7%)	0.039
CHF *n* (%)	20 (11.2%)	24 (22.4%)	0.011
Stroke *n* (%)	8 (5.6%)	12 (14.5%)	0.024
CKD *n* (%)	33 (18.5%)	35 (32.7%)	0.007
COPD *n* (%)	24 (13.5%)	19 (17.8%)	NS
Anemia *n* (%)	34 (19.1%)	20 (18.7%)	NS
Infection *n* (%)	54 (30.3%)	29 (27.1%)	NS
Dementia *n* (%)	25 (14.0%)	23 (21.5%)	NS
NLR	4.2 ± 0.3	5.2 ± 0.4	0.050
Neutrophils (×10^3^/μL)	5.2 ± 0.2	6.5 ± 0.4	<0.01
Lymphocytes (×10^3^/μL)	1.54 ± 0.05	1.52 ± 0.07	NS
SIRI index	2.4 ± 0.9	3.9 ± 0.4	0.050
Fasting Glucose (mg/dL)	93.9 ± 1.6	128.5 ± 5.6	<0.001
Creatinine (mg/dL)	1.14 ± 0.04	1.25 ± 0.06	NS
eGFR, mL/min/1.73 m^2^	58.9 ± 1.7	52.5 ±1.9	0.014
BB-DNA (ng/mL)	19.8 ± 3.5	30.5 ± 4.1	0.012

AF: atrial fibrillation; IHD: ischemic heart disease; CHF: congestive heart failure; CKD: chronic heart disease; COPD: chronic obstructive pulmonary disease; NLR: neutrophil-to-lymphocyte ratio; SIRI: systemic inflammation response index; eGFR: estimated glomerular filtration rate; BB-DNA: circulating bacterial DNA. NS: Not significant.

**Table 2:** Association between epigenetic ages in ND and DM2 patients and BB-DNA.

	ND Patients		DM2 Patients		References
	Beta	*p* Value	Beta	*p* Value	
Age Acceleration Residual	−0.008	0.925	0.191	0.063	[12]
Age Acceleration Grimage	−0.034	0.711	0.023	0.848	[25]
Age Acceleration PhenoAge	−0.058	0.476	0.202	0.045	[26]
DNAmAge	−0.057	0.582	0.157	0.191	[13]
DNAmAge	−0.048	0.682	0.246	0.071	[12]
IEAA	−0.063	0.430	0.087	0.391	[13]
IEAA	−0.055	0.490	0.164	0.105	[12]
DNAmPhenoAge	−0.074	0.468	0.224	0.045	[26]
DNAmAge Skin Blood Clock	−0.017	0.882	0.455	0.004	[27]
EpigeneticAge	−0.113	0.374	0.345	0.035	[28]
DNAm GrimAge *	−0.116	0.394	−0.031	0.487	[25]
DNAm GrimAge2 *	−0.060	0.629	−0.078	0.613	[29]

Linear regression adjusted for age, gender, atrial fibrillation (AF), ischemic heart disease (IHD), chronic heart failure (CHF), chronic kidney disease (CKD), stroke, neutrophil count, and estimated glomerular filtration rate (eGFR). Intrinsic Epigenetic Age Acceleration (IEAA). * Based on real age.

**Table 3:** Stepwise linear regression analysis of BB-DNA and methylation levels of both inflammatory mediators and *CDKN1A/p21*, *CDKN2A/p16*, and *TP53* genes in DM2 patients.

CpG Sites	UCSC_RefGene_Group	Standardized Coefficients—Beta	*p* Value
IFNγ_cg09711238	TSS200	−0.528	<0.001
IFNγ_cg26227465	TSS200	0.265	0.035
IFNγ_cg12640631	TSS1500	−0.182	0.047
TNFα_cg08553327 *	1stExon	0.408	<0.001
TNFα_cg12681001 *	1stExon	−0.352	0.004
IL6_cg05265849	Body	−0.122	<0.01
IL6_cg21785978	Body; TSS200	0.232	0.007
IL6_cg01770232	TSS1500	−0.178	0.031
IL10_cg17067005	Body	−0.192	0.047
IL1β_cg20983042	TSS1500	−0.237	0.010
IL1β_cg07250315	Body	0.619	<0.001
IL1β_cg07935264	TSS200	−0.374	0.002
IL1β_cg15218327	Body	−0.269	0.005
NFKB1_cg27333178	5′UTR	−0.11	<0.001
NFKB1_cg23462257	TSS1500	−0.205	<0.001
NFKB1_cg23898555	Body	0.129	0.011
NFKB1_cg07955720	Body	−0.111	0.028
CRP_cg25257346	Body	−0.477	<0.001
CRP_cg08474603	TSS200	0.102	0.018
CRP_cg24976805	TSS1500	−0.510	<0.001
CRP_cg09267046	Body	0.291	<0.001
CDKN1A/p21_cg13662121	TSS1500	−0.352	<0.001
CDKN1A/p21_cg09774179	Body	0.300	<0.001
CDKN1A/p21_cg17526952 *	-	−0.222	0.006
CDKN1A/p21_cg06827361	Body	−0.228	0.007
CDKN2A/p16_cg27048359	Body	−0.645	<0.001
CDKN2A/p16_cg13601799 *	1stExon; Body	0.217	0.003
CDKN2A/p16_cg23426614 *	TSS200; TSS1500	−0.187	0.011
CDKN2A/p16_cg01694391 *	TSS1500; Body	−0.198	0.010
TP53_cg27105645	5′UTR	−0.267	0.002
TP53_cg10653997	5′UTR	−0.278	<0.001
TP53_cg08691422	5′UTR; TSS1500	−0.239	0.006
TP53_cg09168066	Body; TSS1500; ExonBnd; 5′UTR	−0.303	<0.001
TP53_cg07343727 *	5′UTR; TSS1500; TSS200	−0.232	0.007
TP53_cg02166782 *	TSS1500; 5′UTR; TSS200	0.225	0.008
TP53_cg15206330 *	TSS1500; 5′UTR; TSS200	−0.168	0.047

* Promoter associated. Linear regression was performed correcting for age, gender, atrial fibrillation (AF), ischemic heart disease (IHD), chronic heart failure (CHF), chronic kidney disease (CKD), stroke, neutrophil count, and estimated glomerular filtration rate (eGFR).

**Table 4:** Stepwise linear regression analysis of BB-DNA and gene methylation levels in the epigenetic profile of type 2 diabetes.

CpG Sites	UCSC_RefGene_ Group	Gene Function	Standardized Coefficients	
			Beta	*p* Value
IGF1_cg02823066	Body	Metabolic regulation (glucose and lipid metabolism); growth and development	−0.270	0.004
IGF1_cg25163611 *	TSS1500		−0.319	<0.001
IGF1_cg18504440	1stExon; 5′UTR; Body		−0.219	0.021
PDK4_cg22758834	Body	Regulation of glucose and fatty acid metabolism	−0.215	0.025
PDK4_cg17075888	Body		−0.194	0.042
ABCG1_cg27641007	Body	Regulation of efflux of phospholipids such as sphingomyelin and cholesterol	−0.253	0.006
ABCG1_cg00222799	Body		−0.494	<0.001
ABCG1_cg00177237	Body		−0.607	<0.001
ABCG1_cg20727187	Body		0.260	0.001
ABCG1_cg18382690	Body		−0.451	0.001
ABCG1_cg02494239	5′UTR; Body		0.226	0.002
ABCG1_cg02370100 *	Body		0.258	0.001
UFM1_cg07243519 *	1stExon;5′UTR	Ufmylation (post-transcriptional modification) ER-associated degradation; regulation of transcription	−0.272	0.004
UFM1_cg07350703 *	TSS1500		0.199	0.034
PFKFB2_cg15339972 *	Body; TSS1500	Regulation of glycolysis; expressed in heart	−0.907	<0.001
PFKFB2_cg20198644	Body		−0.346	<0.001
PFKFB2_cg22944368 *	TSS200		−0.245	0.005
PFKFB2_cg05398095 *	TSS1500		0.547	0.039
ARRDC4_cg01088608	Body	Adapter recruiting ubiquitin-protein ligases; possible role in glucose uptake	−0.277	0.004
ARRDC4_cg09442792	3′UTR		−0.196	0.041
SREBF1_cg23155675	Body	Obesity, type 2 diabetes and insulin sensitivity	−0.792	<0.001
SREBF1_cg27407935	Body		0.197	0.004
SREBF1_cg04805065	Body		−0.178	0.005
SREBF1_cg06619462	Body		0.164	0.014
SREBF1_cg14808739 *	TSS1500		0.157	0.014
SREBF1_cg01049850	Body		−0.137	0.031
SREBF1_cg12244055	3′UTR		0.155	0.042
PLAGL1_cg04895233	TSS1500	Transient neonatal diabetes mellitus	−0.502	<0.001
PLAGL1_cg18316621	TSS1500; TSS200 5′UTR		−0.209	0.009
PLAGL1_cg21416120	TSS1500; TSS200 5′UTR		−0.135	0.043
PLAGL1_cg15262884	5′UTR		−0.471	<0.001
PLAGL1_cg01445838	5′UTR		0.366	<0.001
PLAGL1_cg10254692	TSS1500		0.254	0.025
PLAGL1_cg04696964	5′UTR		0.185	0.022
PLAGL1_cg01659632	3′UTR		0.199	0.007
PLAGL1_cg03562868	TSS1500; 5′UTR; TSS200		−0.177	0.034
HEG1_cg20125761	Body	Regulator of heart and vessel formation	−0.369	<0.001
HEG1_cg06477303	Body		−0.342	<0.001
HEG1_cg00213745	Body		0.280	0.002
HEG1_cg16044109	Body		−0.270	0.002
HEG1_cg10294433	Body		−0.229	0.005
OAZ2_cg13262282	Body	Polyamine biosynthesis, type 2 diabetes	−0.459	<0.001
OAZ2_cg05353131 *	TSS200		−0.295	0.005
OAZ2_cg23061600	TSS1500		0.246	0.009
OAZ2_cg14909603	Body		−0.216	0.023
OAZ2_cg24538975	Body		0.267	0.005
OAZ2_cg07031532 *	TSS1500		0.205	<0.05
FAM3C_cg04873577	Body	Type 2 diabetes and non-alcoholic fatty liver disease	−0.351	<0.001
POP7_cg05340629 *	TSS1500	Ribosome biogenesis	0.342	<0.001
POP7_cg04494750	Body		−0.202	0.031
TCEB2_cg02026611	Body	Transcription elongation and cellular senescence	−0.228	0.018
COMMD7_cg23356674	TSS1500	NF-kappa-B complex activity	−0.279	0.004
COMMD7_cg2339673 *	TSS1500		−0.201	0.037
DECR2_cg04571183	Body	Lipid metabolism	−0.561	<0.001
DECR2_cg27315249	TSS1500; 3′UTR		0.449	<0.001
DECR2_cg00481259	TSS1500		−0.253	0.030
DECR2_cg10509880	Body		−0.211	0.012
BSN_cg16885237	Body	Spatial organization of synaptic vesicle cluster	−0.396	<0.001
BSN_cg04381190	3′UTR		−0.296	0.001
BSN_cg13465832	Body		−0.353	<0.001
BSN_cg11216396	Body		0.221	0.027
BSN_cg13444307	Body		−0.228	0.005
BSN_cg19602139	Body		0.208	0.008
PRDX5_cg01708924	3′UTR	Cellular protection against oxidative stress	−0.385	<0.001
FBXO42_cg02207034	5′UTR	Protein–ubiquitin ligases	−0.449	<0.001
FBXO42_cg06216849	Body		−0.219	0.013
FBXO42_cg22937685	Body		−0.185	0.035

* Promoter associated. Linear regression was performed correcting for age, gender, atrial fibrillation (AF), ischemic heart disease (IHD), chronic heart failure (CHF), chronic kidney disease (CKD), stroke, neutrophil count, and estimated glomerular filtration rate (eGFR).

## Data Availability

The data that support the findings of this study are available from the authors upon reasonable request.

## Supplementary Materials

The following supporting information can be downloaded at: https://www.mdpi.com/article/10.3390/ijms26146564/s1.
