# Supplementary material for: Circulating Bacterial DNA as a Novel Blood-Based Biomarker in Type 2 Diabetes Mellitus (DM2): Results from the PROMOTERA Study
Source: Int J Mol Sci. 2025 Jul 8;26(14):6564. doi: 10.3390/ijms26146564 (PMC12294340; doi:10.3390/ijms26146564)
Supplement: Supplementary file 1 [file ijms-26-06564-s001.zip › ijms-3692894-supplementary.pdf]

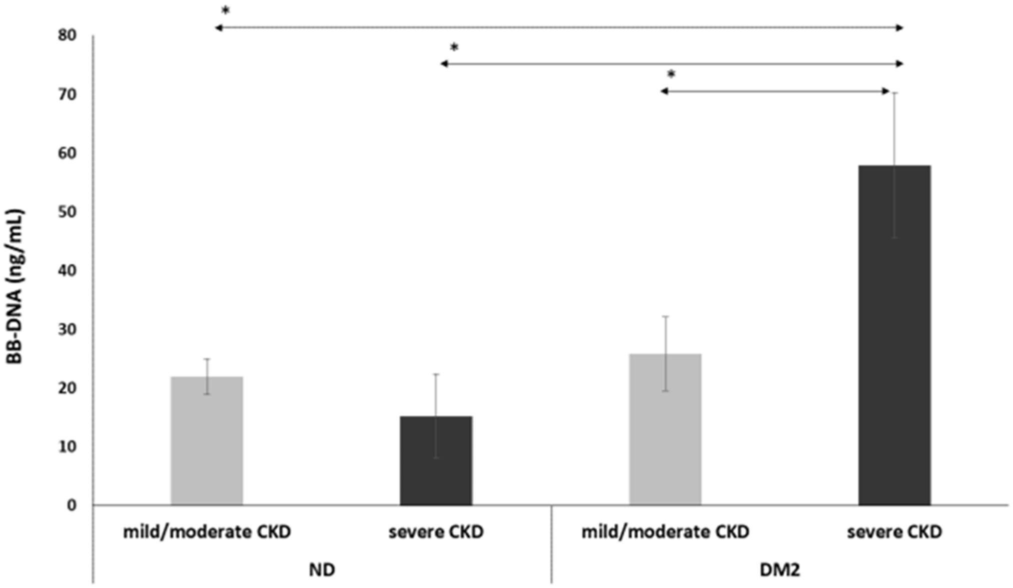


**Figure S1.** Circulating bacterial DNA (BB-DNA) in relation to the chronic kidney disease (CKD) in the PROMOTERA cohort. *p<0.05 compared to DM2 patients with severe CKD. ANCOVA analysis adjusting for age, gender, atrial fibrillation (AF), ischemic heart disease (IHD), chronic heart failure (CHF), stroke, and neutrophil count.

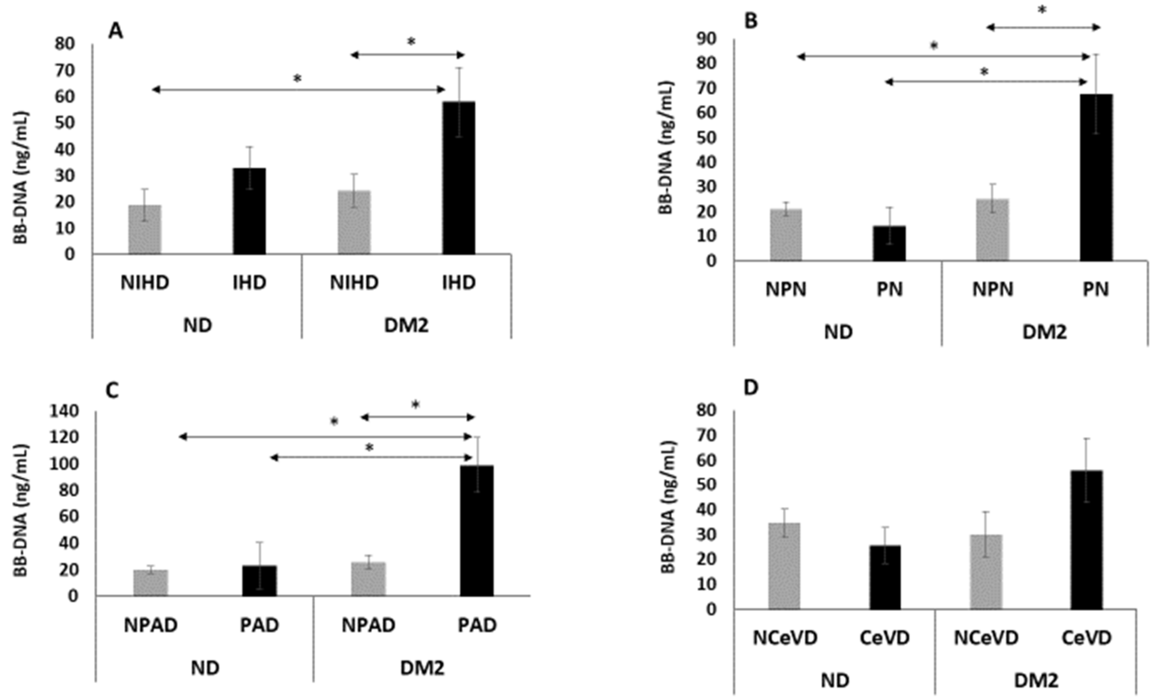


**Figure S2.** Circulating bacterial DNA (BB-DNA) in relation to the diabetic complications in the PROMOTERA cohort. Negative-IHD patients (NIHD), peripheral neuropathy (PN) PN-negative patients (NPN), peripheral artery disease (PAD), PAD-negative patients (NPAD), Cerebrovascular diseases (CeV), CeV-negative patients (NCeV). Among non-diabetic patients, 18 had IHD, 21 PN, 4 PAD, and 43 CeVD; among DM2 patients, 20 had IHD, 13 PN, 10 PAD, and 58 CeVD.*p<0.05. ANCOVA analysis adjusting for age, gender, atrial fibrillation (AF), ischemic heart disease (IHD) (only for panel B, C, D), chronic heart failure (CHF), stroke (only for panel A, B, C), estimated glomerular filtration rate (eGFR) and neutrophil count.

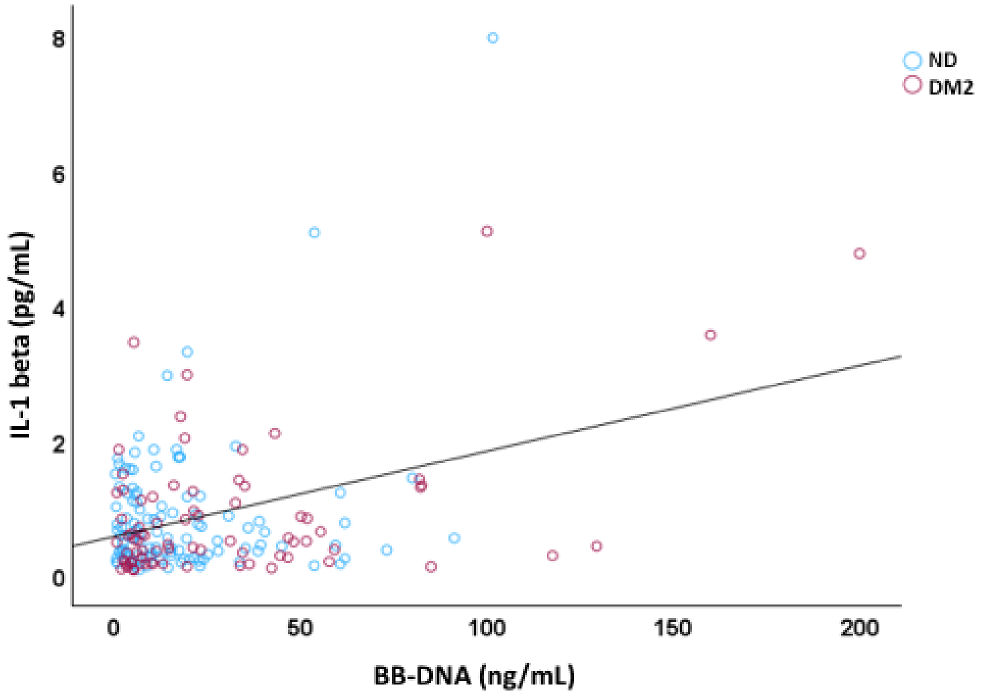


**Figure S3.** Scatter plots and linear regression between BB-DNA and plasma IL-1 beta in the overall PROMOTERA cohort. A positive association between BB-DNA and IL-1 beta was observed (β= 0.252 p=0.003), after correction with age, sex, diabetes (DM2), atrial fibrillation (AF), ischemic heart disease (IHD), chronic heart failure (CHF), chronic kidney disease (CKD), stroke, neutrophil count, and estimated glomerular filtration rate (eGFR).

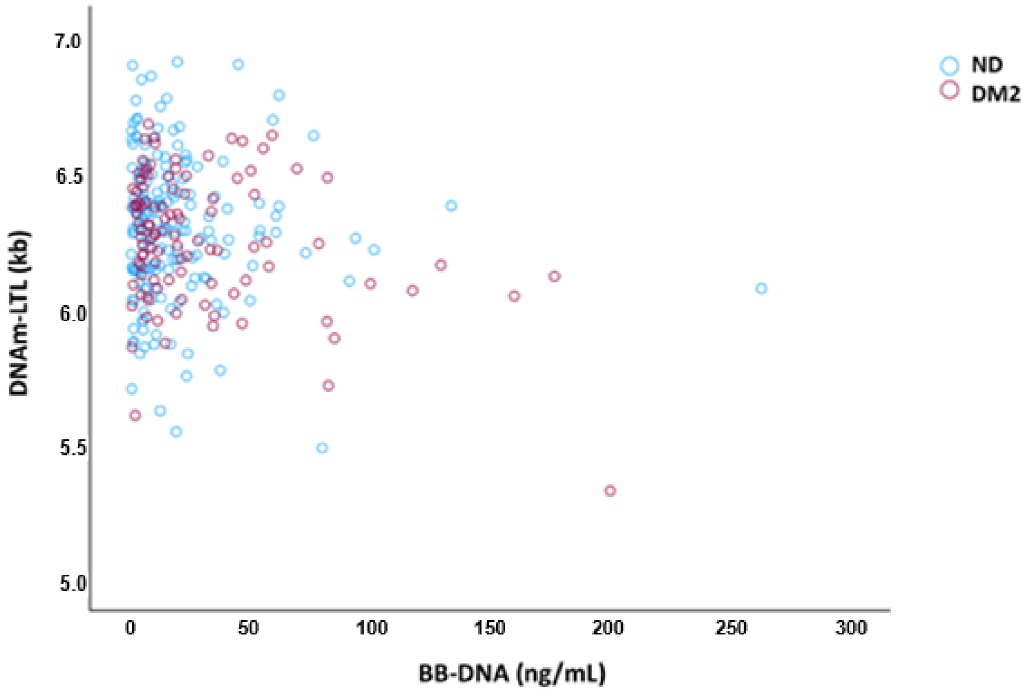


**Figure S4.** Scatter plots and linear regression between BB-DNA and DNAm-LTL in the overall PROMOTERA cohort. A negative association between BB-DNA and DNAm-LTL was observed (β= -0.143 p=0.013), after correction with age, sex, diabetes (DM2) , atrial fibrillation (AF), ischemic heart

disease (IHD), chronic heart failure (CHF), chronic kidney disease (CKD), stroke, neutrophil count, and estimated glomerular filtration rate (eGFR).

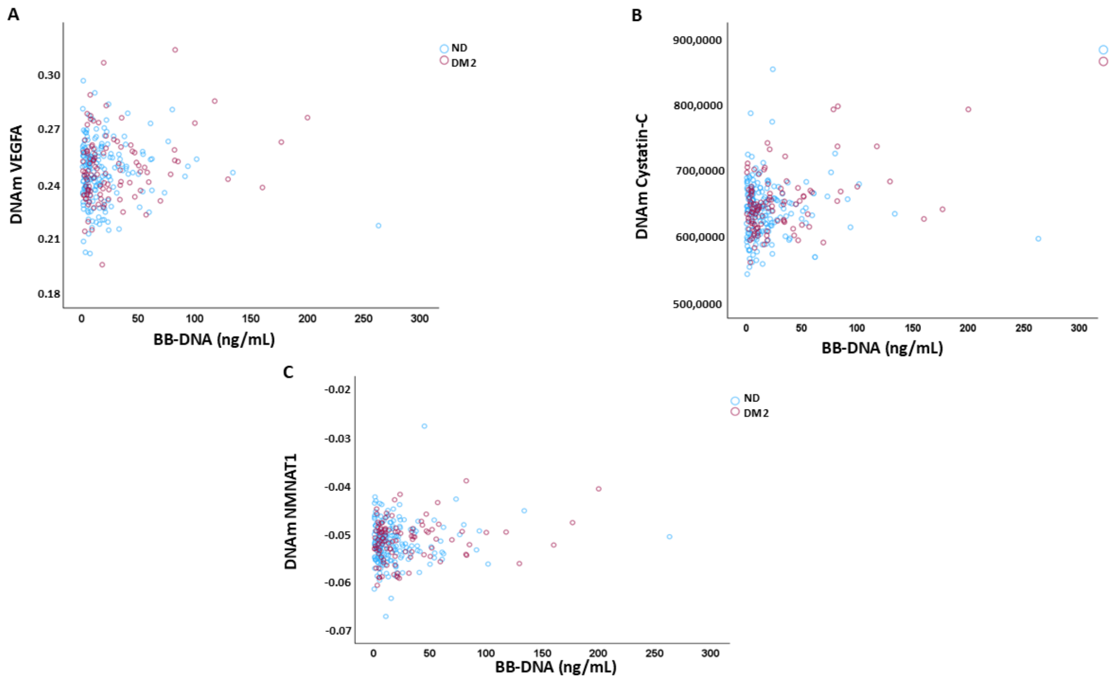


**Figure S5.** Scatter plots and linear regression between BB-DNA, DNAmVEGFA (A), DNAm cystatin C (B) and DNAmNMNAT1 (C) in the overall PROMOTERA cohort. A trend toward a positive association was observed between BB-DNA and plasma DNAmVEGFA ($\beta = 0.085$, $p = 0.121$), as well as DNAm cystatin C ($\beta = 0.173$, $p = 0.072$), while DNAmNMNAT1 showed a significant positive correlation with BB-DNA ($\beta = 0.195$, $p < 0.01$), after adjustment for age, sex, diabetes (DM2), atrial fibrillation (AF), ischemic heart disease (IHD), chronic heart failure (CHF), chronic kidney disease (CKD), stroke, neutrophil count, and estimated glomerular filtration rate (eGFR).

**Table S1.** Linear Regression Analysis of circulating bacterial DNA (BB-DNA) and DNAm-based estimators of pro-inflammatory factors.

| | **ND patients** | | **DM2 patients** | |
|---|---|---|---|---|
| | **Beta** | **P value** | **Beta** | **P value** |
| DNAm CXCL9 | -0.164 | 0.238 | 0.164 | 0.331 |
| DNAm CXCL10 | -0.050 | 0.535 | 0.170 | 0.123 |
| DNAm CXCL11 | -0.104 | 0.148 | -0.095 | 0.350 |
| DNAm CCL11 | 0.080 | 0.351 | -0.008 | 0.938 |
| DNAm IL-18R1 | -0.028 | 0.723 | -0.041 | 0.692 |
| DNAm IL-6 | 0.060 | 0.470 | 0.168 | 0.123 |
| DNAm Beta-2-Microglobulin | -0.052 | 0.663 | 0.259 | 0.055 |
| DNAm Oncostatin M | 0.012 | 0.880 | 0.173 | 0.118 |
| DNAm TNF-β | -0.108 | 0.248 | 0.162 | 0.144 |
| DNAm GDF-15 | -0.095 | 0.279 | 0.154 | 0.161 |
| DNAm PAI-1 | -0.050 | 0.464 | 0.157 | 0.160 |
| DNAm TIMP-1 | 0.013 | 0.895 | 0.229 | 0.086 |
| DNAm leptin | -0.096 | 0.468 | -0.213 | 0.268 |

Linear regression was performed correcting for age, gender, atrial fibrillation (AF), ischemic heart disease (IHD), chronic heart failure (CHF), chronic kidney disease (CKD), stroke, neutrophil count, and estimated glomerular filtration rate (eGFR).

**Table S2.** Linear Regression Analysis of circulating bacterial DNA (BB-DNA) and DNAm-based estimators of blood cell types.

| | ND patients | | DM2 patients | |
|---|---|---|---|---|
| | **Beta** | **P value** | **Beta** | **P value** |
| CD4+T cells | -0.049 | 0.550 | 0.047 | 0.651 |
| CD8+T cells | 0.001 | 0.998 | 0.080 | 0.425 |
| CD4 naiveT cells | -0.120 | 0.121 | 0.070 | 0.492 |
| CD8 naiveT cells | -0.039 | 0.647 | -0.225 | **0.025** |
| NK cells | 0.062 | 0.425 | 0.069 | 0.491 |
| B cells | 0.028 | 0.719 | 0.127 | 0.226 |
| Granulocytes/neutrophils | 0.020 | 0.804 | -0.065 | 0.515 |
| Plasma blasts | 0.021 | 0.795 | -0.191 | 0.095 |
| Monocytes | 0.036 | 0.647 | -0.078 | 0.450 |

Linear regression was performed correcting for age, gender, atrial fibrillation (AF), ischemic heart disease (IHD), chronic heart failure (CHF), chronic kidney disease (CKD), stroke, neutrophil count, and estimated glomerular filtration rate (eGFR).

**Table S3.** Stepwise Linear Regression Analysis of circulating bacterial DNA (BB-DNA) and gene methylation patterns of key Type 2 Diabetes genes in ND patients.

| **CpG sites** | **UCSC_RefGene_ Group** | **Gene function** | **Standardized coef-ficients** | |
|---|---|---|---|---|
| | | | **Beta** | **P value** |
| IGF1_cg16655193 | Body | | -0.230 | 0.001 |
| IGF1_cg16801374 | Body; TSS1500 | | 0.211 | 0.003 |
| IGF1_cg24676120 | Body | | -0.177 | 0.016 |
| ABCG1_ cg12382576 | Body | Regulation of efflux of phospholipids such as sphingomye-lin and cholesterol | -0.227 | 0.002 |
| ABCG1_ cg07875759 | TSS1500; TSS200 5'UTR; Body | | -0.305 | <0.001 |
| ABCG1_ cg01289965 | Body;5'UTR; TSS200 | | 0.266 | 0.001 |
| ABCG1_ cg26519745 | Body;5'UTR; TSS200 | | -0.288 | <0.001 |
| ABCG1_ cg06030219 | TSS1500; TSS200 5'UTR; Body | | 0.195 | 0.006 |
| UFM1_ cg02655108* | 5'UTR; Body | Ufmylation (post-trascriptional modi-fication) ER-Associ-ated Degradation; Regulation of Tran-scription | -0.161 | 0.024 |
| UFM1_ cg18352063* | 1stExon;5'UTR | | -0.177 | 0.027 |
| PFKFB2_cg19080559 | Body | Regulation of Gly-colysis; expressed in heart | -0.275 | <0.001 |
| PFKFB2_cg22944368* | TSS200 | | 0.214 | 0.004 |
| PFKFB2_cg17858100* | TSS200 | | -0.179 | 0.014 |
| ARRDC4_cg09442792 | 3'UTR | | -0.312 | <0.01 |
| SREBF1_cg06261007* | 1stExon;5'UTR | Obesity, type 2 diabe-tes and insulin sensitivity | 0.198 | 0.008 |
| SREBF1_cg09796270 | Body | | -0.212 | 0.006 |
| SREBF1_cg21020221 | Body | | -0.190 | 0.012 |
| SREBF1_cg27407935 | Body | | -0.251 | 0.004 |

| | | | | |
|---|---|---|---|---|
| SREBF1_cg23875758 | 3'UTR | | 0.255 | 0.003 |
| PLAGL1_ cg12757684 | 1stExon; Body; 5'UTR | Transient neonatal diabetes mellitus | 0.208 | 0.005 |
| PLAGL1_ cg20443501 | TSS1500;5'UTR; TSS200 | | -0.285 | <0.001 |
| PLAGL1_ cg01183424 | 5'UTR | | 0.197 | 0.011 |
| HEG1_ cg11029367 | Body | Regulator of heart and vessel formation | -0.176 | 0.022 |
| FAM3C_ cg12833292 | TSS1500 | Type 2 diabetes and non-alcoholic fatty liver disease | 0.162 | 0.034 |
| COMMD7_cg06604199* | TSS200 | NF-kappa-B complex activity | 0.151 | 0.049 |
| BSN_ cg19602139 | Body | Spatial organization of synaptic vesicle cluster | -0.194 | 0.009 |
| BSN_cg13444307 | Body | | -0.181 | 0.035 |
| BSN_ cg22881573 | Body | | 0.157 | 0.028 |
| BSN_cg03049732 | Body | | -0.231 | 0.007 |
| BSN_cg24296397 | Body | | 0.175 | 0.035 |
| FBXO42_ cg14627669 | 3'UTR | Protein-ubiquitin ligases | -0.197 | 0.010 |

*Promoter associated. Linear regression was performed correcting for age, gender, atrial fibrillation (AF), ischemic heart disease (IHD), chronic heart failure (CHF), chronic kidney disease (CKD), stroke, neutrophil count, and estimated glomerular filtration rate (eGFR).

**Table S4.** Association between epigenetic ages and BB-DNA in the overall PROMOTERA cohort.

| | Promotera cohort N=285 | | References |
|---|---|---|---|
| | **Beta** | **P value** | |
| Age Acceleration Residual | 0.105 | 0.140 | 12 |
| Age Acceleration Grimage | -0.002 | 0.978 | 25 |
| Age Acceleration PhenoAge | 0.093 | 0.198 | 26 |
| DNAmAge | 0.070 | 0.416 | 13 |
| DNAmAge | 0.144 | 0.156 | 12 |
| IEAA | 0.035 | 0.509 | 13 |
| IEAA | 0.065 | 0.358 | 12 |
| DNAmPhenoAge | 0.108 | 0.210 | 26 |
| DNAmAge Skin Blood Clock | 0.244 | 0.018 | 27 |
| EpigeneticAge | 0.155 | 0.183 | 28 |
| DNAm GrimAge* | 0.010 | 0.926 | 25 |
| DNAm GrimAge2* | -0.002 | 0.988 | 29 |

Linear regression adjusted for age, gender, diabetes (DM2), atrial fibrillation (AF), ischemic heart disease (IHD), chronic heart failure (CHF), chronic kidney disease (CKD), stroke, neutrophil count, and estimated glomerular filtration rate (eGFR). Intrinsic Epigenetic Age Acceleration (IEAA). * Based on real age.

**Table S5.** Stepwise linear regression analysis of BB-DNA and methylation levels of both inflammatory mediators and CDKN1A/p21, CDKN2A/p16, and TP53 genes in the overall PROMOTERA cohort.

| CpG sites | UCSC_RefGene_Group | Standardized coefficients - Beta | P value |
|---|---|---|---|
| **IFNγ_cg09711238** | TSS200 | -0.236 | <0.001 |
| **IL6_cg01770232** | TSS1500 | -0.127 | 0.029 |
| **IL6_cg21785978** | Body; TSS200 | 0.117 | 0.046 |
| **IL10_cg17067005** | Body | -0.144 | 0.017 |
| **IL1β_cg07250315** | Body | 0.222 | 0.001 |
| **IL1β_cg15218327** | Body | -0.162 | 0.007 |
| **NFKB1_cg27333178** | 5'UTR | -0.265 | <0.001 |
| NFKB1_ cg23655858 | Exon;Body | 0.151 | 0.011 |
| NFKB1_ cg15409712 | 5'UTR | -0.186 | 0.002 |
| **CRP_cg25257346** | Body | -0.298 | <0.001 |
| **CRP_cg24976805** | TSS1500 | -0.301 | <0.001 |
| CRP_cg15076824 | TSS1500 | -0.183 | 0.013 |
| **CDKN1A/p21_cg13662121** | TSS1500 | -0.254 | <0.001 |
| **CDKN1A/p21_cg09774179** | Body | 0.199 | <0.001 |
| **CDKN1A/p21_cg17526952*** | - | -0.324 | 0.006 |
| CDKN1A/p21_ cg01955533 | 5'UTR | -0.221 | 0.006 |
| CDKN2A/p16_ cg21091547 | Body | -0.126 | 0.032 |
| **CDKN2A/p16_cg13601799*** | 1stExon; Body | 0.153 | 0.006 |
| **CDKN2A/p16_cg23426614*** | TSS200; TSS1500 | -0.144 | 0.010 |
| CDKN2A/p16_ cg27048359 | - | -0.394 | 0.001 |
| **TP53_cg08691422** | 5'UTR; TSS1500 | -0.157 | 0.008 |
| **TP53_cg09168066** | Body; TSS1500; ExonBnd; 5'UTR | -0.185 | 0.002 |
| TP53_ cg02166782* | TSS1500; 5'UTR; TSS200 | 0.117 | 0.048 |

* Promoter associated. Linear regression was performed correcting for age, gender, diabetes (DM2), atrial fibrillation (AF), ischemic heart disease (IHD), chronic heart failure (CHF), chronic kidney disease (CKD), stroke, neutrophil count, and estimated glomerular filtration rate (eGFR). CpG sites initially associated with BB-DNA in DM2 patients and confirmed in the analysis of the entire PROMOTERA cohort are shown in bold.

**Table S6.** Stepwise Linear Regression analysis of BB-DNA and gene methylation levels in the epigenetic profile of Type 2 Diabetes in the overall PROMOTERA cohort.

| CpG sites | UCSC_RefGene_Group | Gene function | Standardized coefficients | |
|---|---|---|---|---|
| | | | Beta | P value |
| **IGF1_cg02823066** | Body | Metabolic Regulation (glucose and lipid metabolism); Growth and Development | -0.293 | <0.01 |
| **IGF1_cg25163611*** | TSS1500 | | -0.156 | 0.014 |
| IGF1_ cg01305421 | 5'UTR;1stExon | | -0.218 | 0.004 |
| **PDK4_cg22758834** | Body | Regulation of glucose and fatty acid metabolism | -0.149 | 0.038 |
| ABCG1_ cg16740586 | Body | Regulation of efflux of phospholipids such as sphingomyelin and cholesterol | -0.306 | <0.001 |
| ABCG1_ cg20973598 | Body | | 0.410 | <0.001 |
| ABCG1_ cg12382576 | Body | | -0.268 | <0.001 |
| **ABCG1_cg02494239** | 5'UTR; Body | | 0.235 | 0.001 |
| **ABCG1_cg00177237** | Body | | -0.356 | 0.003 |
| **ABCG1_cg27641007** | Body | | -0.169 | 0.016 |
| ABCG1_ cg21202699 | Body | | 0.156 | 0.014 |

| | | | | |
|---|---|---|---|---|
| ABCG1_ cg07875759 | TSS1500; TSS200 5'UTR; Body | | -0.151 | 0.016 |
| ABCG1_ cg17526396 | 5'UTR | | 0.145 | 0.032 |
| **ABCG1_cg20727187** | Body | | 0.122 | 0.048 |
| **UFM1_cg07243519*** | 1stExon;5'UTR | Ufmylation (post-trascriptional modification) ER-Associated Degradation; Regulation of Transcription | -0.184 | 0.005 |
| UFM1_ cg13452070* | TSS1500 | | -0.163 | 0.014 |
| **PFKFB2_cg15339972*** | Body; TSS1500 | Regulation of Glycolysis; expressed in heart | -0.429 | <0.01 |
| PFKFB2_ cg16932062 | TSS1500;5'UTR;ExonBnd | | -0.273 | 0.001 |
| PFKFB2_ cg19080559 | Body | | -0.149 | 0.022 |
| **ARRDC4_cg09442792** | 3'UTR | | -0.222 | <0.001 |
| **SREBF1_cg23155675** | Body | Obesity, type 2 diabetes and insulin sensitivity | -0.164 | <0.01 |
| SREBF1_ cg19619576 | TSS1500;Body | | -0.211 | 0.001 |
| SREBF1_ cg25999891 | Body | | 0.150 | 0.015 |
| **SREBF1_cg06619462** | Body | | 0.151 | 0.020 |
| SREBF1_ cg06261007 | 1stExon;5'UTR | | 0.211 | 0.001 |
| SREBF1_ cg20544516 | Body | | 0.249 | 0.001 |
| SREBF1_ cg03164243 | TSS1500;Body | | -0.245 | 0.002 |
| **PLAGL1_cg04895233** | TSS1500 | Transient neonatal diabetes mellitus | -0.176 | <0.01 |
| **PLAGL1_cg18316621** | TSS1500; TSS200 5'UTR | | -0.148 | 0.027 |
| PLAGL1_ cg13607311 | 3'UTR | | -0.190 | 0.002 |
| **PLAGL1_cg15262884** | 5'UTR | | -0.307 | <0.001 |
| **PLAGL1_cg01445838** | 5'UTR | | 0.302 | <0.001 |
| PLAGL1_ cg10717379 | 5'UTR | | -0.341 | <0.001 |
| PLAGL1_ cg09307468 | TSS1500 | | 0.136 | 0.027 |
| **PLAGL1_ cg01659632** | 3'UTR | | 0.144 | 0.017 |
| PLAGL1_cg18316621 | 5'UTR | | -0.148 | 0.019 |
| **HEG1_cg20125761** | Body | Regulator of heart and vessel formation | -0.175 | <0.01 |
| HEG1_ cg25680412 | 3'UTR | | -0.219 | <0.001 |
| HEG1_ cg14706455 | TSS1500 | | 0.176 | 0.012 |
| **HEG1_cg16044109** | Body | | -0.220 | 0.003 |
| HEG1_ cg07313613 | Body | | 0.149 | 0.024 |
| **OAZ2_cg13262282** | Body | Polyamine biosynthesis, type 2 diabetes | -0.201 | 0.003 |
| **OAZ2_cg05353131*** | TSS200 | | -0.188 | 0.007 |
| **OAZ2_cg24538975** | Body | | 0.162 | 0.017 |
| **OAZ2_ cg07031532*** | TSS1500 | | 0.144 | 0.042 |
| **FAM3C_cg04873577** | Body | Type 2 diabetes and non-alcoholic fatty liver disease | -0.208 | 0.002 |
| **POP7_cg05340629*** | TSS1500 | Ribosome biogenesis | 0.206 | 0.002 |
| **TCEB2_cg02026611** | Body | Transcription elongation and cellular senescence | -0.192 | 0.006 |
| COMMD7_cg23356674 | TSS1500 | NF-kappa-B complex activity | -0.154 | 0.023 |
| **DECR2_cg04571183** | Body | Lipid metabolism | -0.377 | <0.001 |
| **DECR2_cg27315249** | TSS1500;3'UTR | | 0.356 | 0.001 |
| **DECR2_cg00481259** | TSS1500 | | -0.205 | 0.038 |
| **BSN_cg16885237** | Body | Spatial organization of synaptic vesicle cluster | -0.392 | <0.001 |
| BSN_cg22881573 | Body | | 0.184 | 0.004 |
| **PRDX5_cg01708924** | 3'UTR | Cellular protection against oxidative | -0.196 | 0.003 |
| **PRDX5_cg14270124*** | TSS200 | | 0.154 | 0.019 |

| | | | | |
|---|---|---|---|---|
| | | stress | | |
| **FBXO42_cg02207034** | 5'UTR | Protein-ubiquitin ligases | -0.227 | <0.001 |
| FBXO42_cg23281809 | 5'UTR | | -0.173 | 0.008 |
| FBXO42_ cg05059632 | 5’UTR | | 0.153 | 0.018 |
| FBXO42_ cg03978675* | TSS1500 | | -0.142 | 0.027 |

*Promoter associated. Linear regression was performed correcting for age, gender, diabetes (DM2), atrial fibrillation (AF), ischemic heart disease (IHD), chronic heart failure (CHF), chronic kidney disease (CKD), stroke, neutrophil count, and estimated glomerular filtration rate (eGFR). CpG sites initially associated with BB-DNA in DM2 patients and confirmed in the analysis of the entire PROMOTERA cohort are shown in bold.

**Table S7.** Linear Regression Analysis of circulating bacterial DNA (BB-DNA) and DNAm-based estimators of pro-inflammatory factors in the overall PROMOTERA cohort.

| | **Promotera cohort N=285** | |
|---|---|---|
| | **Beta** | **P value** |
| DNAm CXCL9 | 0.020 | 0.770 |
| DNAm CXCL10 | -0.026 | 0.710 |
| DNAm CXCL11 | .0.096 | 0.116 |
| DNAm CCL11 | 0.009 | 0.892 |
| DNAm IL-18R1 | -0.032 | 0.650 |
| DNAm IL-6 | -0.039 | 0.570 |
| DNAm Beta-2-Microglobulin | 0.024 | 0.617 |
| DNAm Oncostatin M | 0.010 | 0.878 |
| DNAm TNF-β | -0.031 | 0.583 |
| DNAm GDF-15 | -0.054 | 0.346 |
| DNAm PAI-1 | -0.120 | 0.067 |
| DNAm TIMP-1 | 0.012 | 0.572 |
| DNAm leptin | -0.085 | 0.062 |

Linear regression was performed correcting for age, gender, diabetes (DM2), atrial fibrillation (AF), ischemic heart disease (IHD), chronic heart failure (CHF), chronic kidney disease (CKD), stroke, neutrophil count, and estimated glomerular filtration rate (eGFR).

**Table S8.** Linear Regression Analysis of circulating bacterial DNA (BB-DNA) and DNAm-based estimators of blood cell types in the overall PROMOTERA cohort.

| | **Promotera cohort N=285** | |
|---|---|---|
| | **Beta** | **P value** |
| CD4+T cells | 0.022 | 0.745 |
| CD8+T cells | 0.076 | 0.261 |
| CD4 naiveT cells | -0.070 | 0.328 |
| CD8 naiveT cells | -0.138 | **0.045** |
| NK cells | 0.159 | 0.057 |
| B cells | 0.065 | 0.370 |
| Granulocytes/neutrophils | -0.077 | 0.240 |
| Plasma blasts | -0.111 | 0.106 |
| Monocytes | -0.100 | 0.165 |

Linear regression was performed correcting for age, gender, diabetes (DM2), atrial fibrillation (AF), ischemic heart disease (IHD), chronic heart failure (CHF), chronic kidney disease (CKD), stroke, neutrophil count, and estimated glomerular filtration rate (eGFR).
